# Anti-CSF-1 treatment is effective to prevent carcinoma invasion induced by monocyte-derived cells but scarcely by microglia

**DOI:** 10.18632/oncotarget.3855

**Published:** 2015-05-12

**Authors:** Eva Rietkötter, Annalen Bleckmann, Michaela Bayerlová, Kerstin Menck, Han-Ning Chuang, Britta Wenske, Hila Schwartz, Neta Erez, Claudia Binder, Uwe-Karsten Hanisch, Tobias Pukrop

**Affiliations:** ^1^ Department of Hematology and Medical Oncology, University Medical Center, 37075 Göttingen, Germany; ^2^ Department of Medical Statistics, University Medical Center, 37075 Göttingen, Germany; ^3^ Department of Pathology, Sackler School of Medicine, 69978 Tel Aviv University, Israel; ^4^ Institute of Neuropathology, University Medical Center, 37075 Göttingen, Germany; ^5^ Department of Hematology and Medical Oncology, University Clinic Regensburg, 93053 Regensburg, Germany

**Keywords:** anti-CSF-1, colonization, monocyte-derived cells, microglia, metastasis

## Abstract

The mononuclear phagocytic system is categorized in three major groups: monocyte-derived cells (MCs), dendritic cells and resident macrophages. During breast cancer progression the colony stimulating factor 1 (CSF-1) can reprogram MCs into tumor-promoting macrophages in the primary tumor. However, the effect of CSF-1 during colonization of the brain parenchyma is largely unknown.

Thus, we analyzed the outcome of anti-CSF-1 treatment on the resident macrophage population of the brain, the microglia, in comparison to MCs, alone and in different *in vitro* co-culture models. Our results underline the addiction of MCs to CSF-1 while surprisingly, microglia were not affected. Furthermore, in contrast to the brain, the bone marrow did not express the alternative ligand, IL-34. Yet treatment with IL-34 and co-culture with carcinoma cells partially rescued the anti-CSF-1 effects on MCs. Further, MC-induced invasion was significantly reduced by anti-CSF-1 treatment while microglia-induced invasion was reduced to a lower extend. Moreover, analysis of lung and breast cancer brain metastasis revealed significant differences of CSF-1 and CSF-1R expression.

Taken together, our findings demonstrate not only differences of anti-CSF-1 treatment on MCs and microglia but also in the CSF-1 receptor and ligand expression in brain and bone marrow as well as in brain metastasis.

## INTRODUCTION

Up to thirty percent of breast cancer patients with metastatic disease suffer from brain metastasis, resulting in very unfavorable prognosis even in comparison to metastatic disease in other sites [1]. To make matters worse, hardly any progress has been made over the last decades in the treatment modalities and (animal) models to treat or study brain metastasis [2–4].

This is partially due to the hypothesis of the *linear progression model* of metastasis, were a successful treatment of the primary tumor should inhibit seeding to distant organs [5]. Consistent with this model, the majority of clinical trials as well as animal experiments were performed to prevent or treat the primary tumor but fewer to understand the colonization of distant organs.

It is now accepted that in most cases the distant organs are already seeded at the time when the primary tumor is diagnosed (*parallel progression or early dissemination model*) [6]. Thus, even an effective treatment of the primary tumor cannot inhibit metastatic seeding. Additionally, treatment response of the primary tumor cannot be equated with response of the already seeded metastatic cells in distant organs, due to the unique characteristics of distinct microenvironments. In particular, metastatic cells that disseminated and seeded the brain are already protected by the blood-brain-barrier (BBB) and the specific of the brain, which includes the microglia [7]. Following invasion and seeding, colonization is the crucial step for the formation of macroscopic metastasis [8–10]. In order to characterize the mechanisms that underlie metastatic colonization, it is important to study the specific microenvironments of individual metastatic host organs. The brain has a unique defense system composed of microglia and astrocytes [11]. Recently we and others demonstrated that this organ-specific cellular defense is activated by intruding cancer cells [12, 13]. Importantly, while this glial-defense is potent to induce apoptosis and to eliminate benign epithelial cells, the carcinoma cells do not only sustain this defense but in fact they misuse it [14]. For example, microglia serve as cellular transporters for tumor cells and foster their invasion [13], and depletion of microglia by bisphosphonates reduces this glial-induced invasion [13, 15]. However, depletion of microglia by bisphosphonates also results in reduced glial-induced apoptosis and therefore weakens their defense capacity [14]. Thus, manipulation rather than depletion of microglia could be a better therapeutic approach.

Colony stimulated factor-1 (CSF-1) could serve as a possible target, known to be part of a paracrine loop between carcinoma cells and blood-derived macrophages (MCs) [16]. This paracrine loop reprograms MCs into tumor-promoting monocyte-derived macrophages and enhances the metastatic capacity of cancer cells in the primary tumor [17]. The significant impact of CSF-1 is well demonstrated in the spontaneous metastasizing MMTV-PymT-transgenic mouse model of breast cancer. In this model, the metastatic capacity is reduced after crossing with CSF-1^op/op^ mice [18]. In the CSF-1^op/op^ model MCs are absent, but microglia are still present, although with reduced numbers [19]. However, microglia revealed a phenotype switch in the CSF-1^op/op^ as well as in CSF-1 overexpressing mice [19–21].

This astonishing discordant observation of MCs and microglia could be explained by current findings: Resident microglia were shown to be derived from yolk sac progenitor cells during embryonic development, even before the formation of hematopoietic stem cells [22, 23]. These new findings are in contrast to the previous notion, by which microglia and other resident macrophages are all derived from MCs. The second finding was the description of an alternative ligand for CSF-1 receptor (CSF-1R), interleukin 34 (IL-34) [24]. These and additional novel findings led to a new classification of the mononuclear phagocyte system (MPS). This classification divides the MPS according their ontogeny, function, location and/or morphology in i) resident macrophages (Kupffer cells, Langerhans cells, alveolar macrophages and microglia), ii) MCs, and iii) dendritic cells (DCs) [25].

In terms of metastasis, these findings have severe consequences because the most affected metastatic organs possess these specialized resident macrophage populations, such as Kupffer cells (in liver), alveolar macrophages (in lung) and microglia (in brain). Directly following extravasation, disseminated metastatic tumor cells encounter these unique resident macrophages whose main task is the protection and homeostasis of their respective organs (organ-specific defense systems). Additionally, these macrophage populations do not only differ from MCs, they also demonstrate distinct organ-specific characteristics. However, the functional interactions of metastatic cells with alveolar macrophages, Kupffer cells or microglia are largely unknown.

The *parallel progression model* and the description of these unique macrophage populations led us to focus on microglia during cerebral metastasis to identify possible therapeutic targets and to prevent metastatic colonization. In view of the CSF-1 paracrine loop and its effects on MCs in the primary tumor, we set out to evaluate whether CSF-1 could be a therapeutic target during colonization of the brain parenchyma.

Here we show that microglia are significantly different from MCs, in particular as to their CSF-1 dependency. Further, the expression of the alternative ligand IL-34 is organ specific and carcinoma cells produce significant amounts of CSF-1 as well as IL-34, which partially interferes with the anti-CSF-1 treatment effects.

## RESULTS

### Anti-CSF-1 antibody 5A1 does not exert cytotoxic effects on breast cancer cells but on macrophages

**Figure 1:**
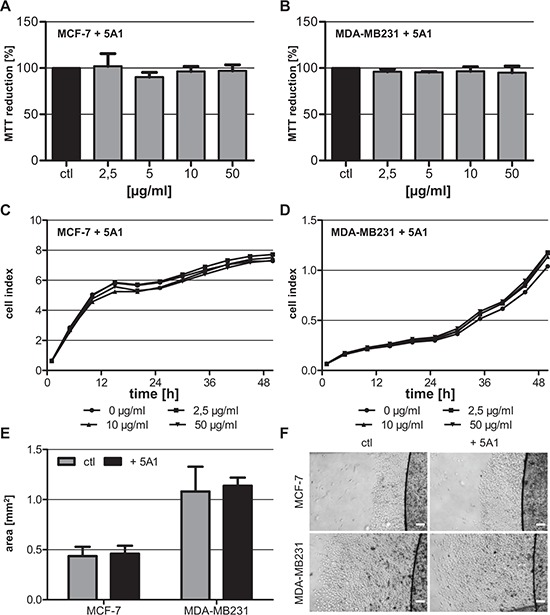
Anti-CSF-1 antibody 5A1 does not exert cytotoxic effects on tested breast cancer cells **A, B.** Metabolic activity of MCF-7 (A) and MDA-MB231 (B) was analyzed 96 h after treatment with 5A1 by measuring MTT reduction (mean ± SD, *n* ≥ 6). **C, D.** MCF-7 (C) and MDA-MB231 (D) were treated with 0 μg/ml (circle), 2.5 μg/ml (square), 10 μg/ml (triangle) and 50 μg/ml (inverse triangle) 5A1. Cell proliferation was measured over 48 h using the xCELLigence system and is indicated as cell index. **E, F.** ECM-based migration assays for MCF-7 and MDA-MB231 over 48 h in the absence (gray bars, left pictures) and presence (black bars, right pictures) of 25 μg/ml 5A1 (mean ± SD, *n* = 4). Scale bars indicate 200 μm.

In this study we sought to analyze the effects of an anti-CSF-1 antibody (clone 5A1) on MC-induced invasiveness of breast cancer cells. To this end, we first determined a concentration of 5A1, which affected the MCs but did not influence the breast carcinoma cells. The human breast cancer cell lines MCF-7 and MDA-MB231 were treated with increasing concentrations of 5A1 for 96 h followed by analysis of metabolic cell activity by MTT-conversion. Both cell lines did not show a reduction in their metabolic activity even at the highest concentration tested (Figure 1A, B). In line with this, proliferation of MCF-7 and MDA-MB231 was also not inhibited by treatment with the anti-CSF-1 antibody (Figure 1C, D). A hallmark characteristic of metastasizing carcinoma cells is their capacity to migrate. To assess whether 5A1 treatment would affect the migration capacity of carcinoma cells we performed ECM-based migration assays. As illustrated in Figure 1E and 1F, both cell lines revealed the same migration pattern following treatment with 5A1.

**Figure 2:**
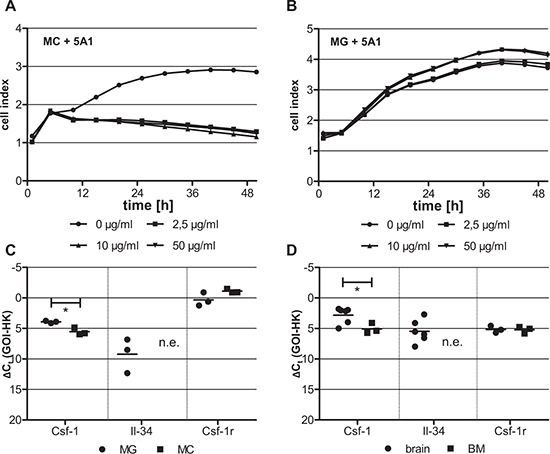
Differing cytotoxicity of 5A1 on distinct macrophage populations is correlated with differing growth factor expression MC **A.** and MG **B.** were treated with 0 μg/ml (circle), 2.5 μg/ml (square), 10 μg/ml (triangle) and 50 μg/ml (inverse triangle) 5A1. Cell proliferation was measured over 48 h using the xCELLigence system and is indicated as cell index. Shown is one representative result (*n* = 3). **C, D.** qRT-PCR for CSF-1, IL-34 and its receptor CSF-1R in (C) MG (circle) and MC (square) and (D) the respective tissue of origin of these two macrophage populations, i.e. brain (circle) and bone marrow (BM, square) (GOI = gene of interest, HK = housekeeper, n.e. = not expressed, *P < 0.05).

CSF-1 is an essential growth factor during the differentiation of myeloid progenitor cells. We thus speculated that MCs would be more sensitive to depletion of CSF-1. To address this question we treated MCs and microglia (MG), with increasing concentrations of the anti-CSF-1 antibody and determined the rate of cell proliferation using the xCELLigence system. As expected, proliferation of MCs was inhibited already at the lowest antibody concentration tested (Figure 2A). In contrast, 5A1 did not inhibit the proliferation of MG (Figure 2B). As shown by calcein-AM /PI-staining reduced proliferation of MCs was not caused by a growth arrest but by increased apoptosis of the cells (Figure S1A). Accordingly, apoptosis of MG was not detectable after 5A1 treatment (Figure S1B).

### IL-34 is differentially expressed in brain and bone marrow

Recently it was shown that IL-34 is an alternative ligand for CSF-1R [24]. We therefore speculated that IL-34 can function as a growth factor for MG but not for MCs, which can explain the opposite effects of 5A1 on cell proliferation of these two macrophage populations. To test this hypothesis, we analyzed the expression levels of CSF-1, IL-34 and CSF-1R not only in the two cell populations but also in the surrounding physiological microenvironment, i.e. the brain and the bone marrow (BM), respectively. We show that CSF-1 is expressed in significantly lower concentrations in MCs and BM as compared to MG and the brain tissue, whereas the expression of CSF-1R is comparable in both cell populations and the respective microenvironments. In contrast, the expression of IL-34 varied dramatically. While IL-34 was expressed in MG as well as in the brain microenvironment, it was not detectable in MCs and BM, respectively (Figure 2C, D). This finding strengthens the hypothesis that MCs might be dependent on CSF-1, whereas MG can compensate for the effects of CSF-1 depletion via the presence of the alternative ligand IL-34.

**Figure 3:**
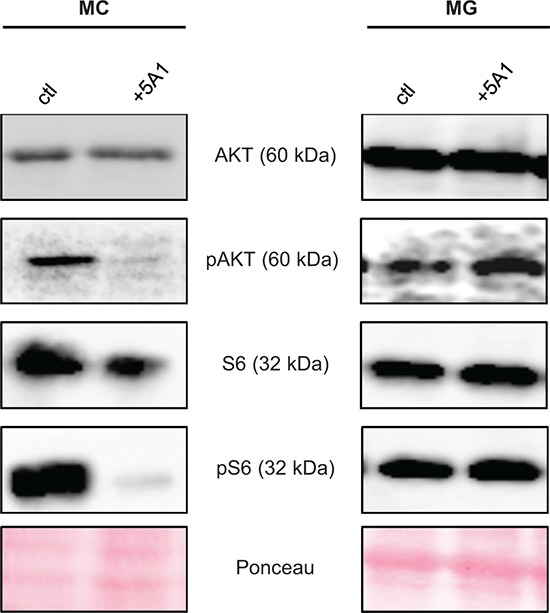
CSF-1R downstream-signaling is affected by 5A1 in MC but not in MG MC (left panel) and MG (right panel) were treated with 2.5 μg/ml 5A1, respectively. Expression of AKT, pAKT, S6 and pS6 was analyzed by western blot after 30 min of treatment. All western blots were repeated at least three times, shown is one representative example.

We next asked whether the varying effect of 5A1 on the viability of MCs and MG can be further explained by impairing the downstream signaling of CSF-1R, which is mediated by AKT and S6. Therefore, we analyzed the expression of these two proteins and their activated (phosphorylated) forms pAKT and pS6 in MCs and MG in the presence or absence of 5A1 by western blot. Results revealed that the anti-CSF-1 antibody does not alter the expression of AKT and S6 whereas the activation of AKT and S6 is inhibited by 5A1 in MCs but not in MG (Figure 3). This finding underlines that MCs, but not MG depend on CSF-1.

### 5A1 decreases macrophage-induced tumor cell invasion

**Figure 4:**
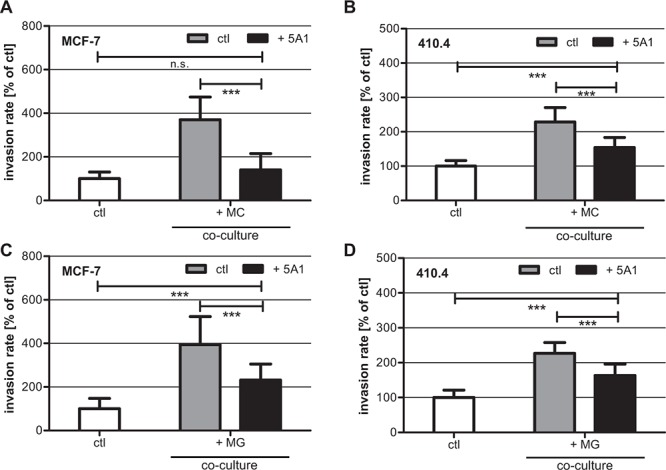
5A1 decreases MG- and MC-induced invasiveness of MCF-7 and 410.4 cells Microinvasion assay of tumor cells alone (white bars) and in co-culture with MC **A, B.** or MG **C, D.** in the absence (gray bars) or presence (black bars) of 2.5 μg/ml 5A1. MC- and MG-induced invasiveness of both MCF-7 (A), (C) and 410.4 (B), (D) is significantly decreased by 5A1. However MG-induced invasion is blocked by 5A1 to a lower extent. Invasiveness is indicated as the percentage of the control tumor cells alone (mean ± SD, *n* ≥ 4, *P < 0.05, ***P < 0.001, n.s. = not significant).

In our previous studies, we demonstrated that MCs induce breast cancer cell invasion [26, 27]. Furthermore, it was shown that this interaction is mediated by a CSF-1/EGF-loop between carcinoma cells and MCs [17]. Therefore, we tested whether a depletion of CSF-1 by the 5A1 antibody would impact MC-induced invasiveness. Thus, we performed modified Boyden chamber assays where we co-cultured human MCF-7 cells and murine 410.4 cells with murine MCs in the presence of 2,5 μg/ml 5A1. As expected MC-induced invasion was significantly reduced following treatment with 5A1 (Figure 4A, B).

In parallel, we tested the effects on MG-induced invasion [13] under the same conditions. A comparable effect was detected when carcinoma cells were co-cultured with MG, although to a significant lower extent (Figure 4C, D). Thus, although 5A1 did not interfere with metabolic activity, viability, migration capacity, or CSF-1R down-stream signalling, it functionally inhibited MG-induced invasiveness of human and murine breast cancer cells.

### Rescue of the 5A1 effect

**Figure 5:**
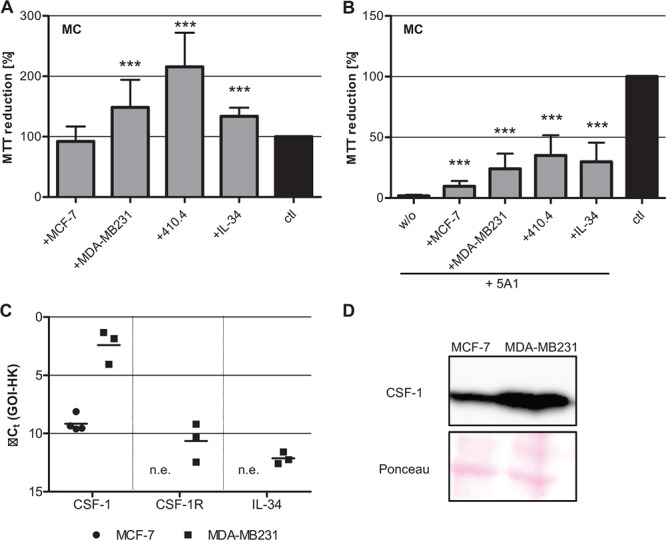
Toxicity of 5A1 on MC can be rescued **A.** Metabolic activity of MC is increased in co-culture with breast cancer cells or IL-34 treatment, analyzed by MTT reduction (mean ± SD, *n* = 4, ***P < 0.001). **B.** Co-culture of MC with tumor cells or treatment with IL-34 partially rescues the toxic effect of 5A1, indicated by MTT-reduction (mean ± SD, *n* = 4; ***P < 0.001, w/o = without additional treatment). **C.** qRT-PCR for the growth factors CSF-1 and IL-34 and their corresponding receptor CSF-1R in MCF-7 (circle) and MDA-MB231 (square) (GOI = gene of interest, HK = housekeeper, n.e. = not expressed). **D.** Protein expression of CSF-1 in MCF-7 and MDA-MB231 analyzed by western blot.

Based on our finding that viability of MG was not affected by 5A1, and the fact that expression of the alternative growth factor IL-34 was detectable in brain but not in bone marrow, we next determined whether the toxic effect of 5A1 on MCs could be rescued by CSF-1 or IL-34. Accordingly, we first analyzed the metabolic activity of MCs in co-culture with carcinoma cells as well as after treatment with IL-34. Interestingly, while co-culture with MCF-7 cells did not alter the metabolic activity of MCs, co-cultivation with MDA-MB231 or 410.4, as well as treatment with IL-34 significantly increased the metabolic activity of macrophages (Figure 5A). This increase in metabolic activity was detectable even following treatment with 5A1, implying a rescue of the anti-CSF-1 effect (Figure 5B), and further suggesting a function for IL-34 as an alternative ligand for CSF-1R. Analysis of the MTT assays revealed that 5A1 toxicity was not only rescued by co-culture with MCF-7, MDA-MB231 or 410.4 but also that the antagonizing effect of the antibody was more pronounced in MDA-MB231 as compared to MCF-7. To clarify if this could be explained by a differential expression of growth factors in these cell lines, we analyzed the expression levels of CSF-1, CSF-1R and IL-34. Indeed, MDA-MB231 cells showed significantly increased expression of CSF-1 as compared with MCF-7 cells not only at the mRNA level, but also when analyzing protein levels (Figure 5C, D). Additionally, MDA-MB231 cells showed a lower expression of CSF-1R and IL-34, while expression of both was not detectable in MCF-7 cells (Figure 5C). These findings may indicate that the highly metastatic basal-like MDA-MB231 cells are more potent in reprogramming the MCs as compared to the less aggressive luminal A MCF-7 breast cancer cell line.

### Differential expression of CSF-1, CSF-1R and IL-34 in brain metastases

**Figure 6:**
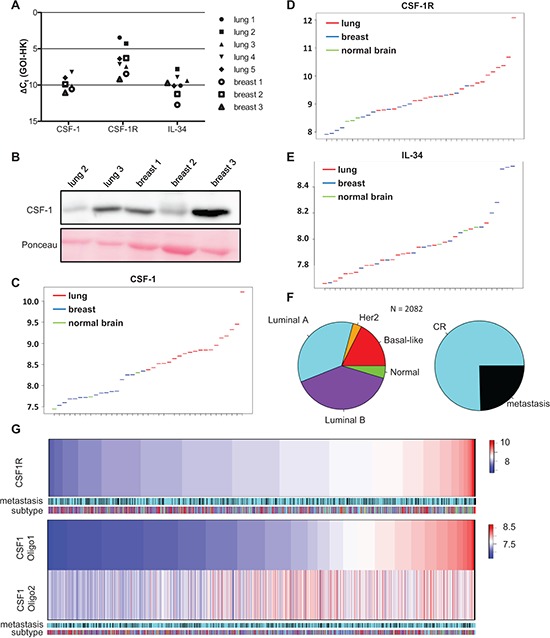
Primary breast cancer and brain metastases differ in their expression of CSF-1, CSF1-R and IL-34 **A.** qRT-PCR for CFS-1, CSF-1R and IL-34 in brain metastases arising from lung carcinoma (black filled symbols) and mamma carcinoma (white filled symbols) (GOI = gene of interest, HK = housekeeper). **B.** CSF-1 expression in five distinct brain metastases analyzed by western blot. **C–E.** Expression levels of (C) CSF-1, (D) CSF-1R and **(E)** IL-34 in brain metastases of lung (red) and mamma carcinoma (blue) as well as in healthy brain tissue (green). **F.** Microarray data sets of *n* = 2082 breast cancer primaries divided into their molecular subtypes (by Genefu) and metastasis free (complete remission = CR) and event of metastasis. **G.** In this cohort of *n* = 2082 patient samples the gene expression of CSF-1R and CSF-1 (two representative oligos) are shown. Neither CSF-1 nor CSF-1R correlated with one molecular subtype or event of metastasis.

In order to validate the relevance of our findings in human disease, and to test whether CSF-1 depletion could potentially be used for the prevention or treatment of brain metastasis, we examined the expression of the growth factors CSF-1 and IL-34 and the corresponding receptor CSF-1R in brain metastases of cancer patients. We performed qRT-PCR analysis on five brain metastases from lung cancer patients and three brain metastases from breast cancer patients. Analysis of the results revealed that CSF-1, IL-34 and CSF-1R were expressed in all samples although the expression levels for a distinct gene varied. For CSF-1 the difference between the sample with the lowest expression and the sample showing the highest expression was 8-fold. For CSF-1R and IL-34 this difference was 64-fold and 32-fold, respectively (Figure 6A). Differential expression of CSF-1 was confirmed also on protein level (Figure 6B).

To further underline the significance of the above findings in a larger dataset we investigated the gene expression of CSF-1, CSF-1R and IL-34 in a cohort of brain metastases of already published microarray gene sets (15 breast brain metastases, 19 lung adenocarcinoma brain metastasis and 3 control brain samples) and compared them for differential gene expression of CSF-1, CSF-1R and IL-34. These analyses revealed CSF-1 to be predominantly expressed in lung brain metastases and to a lesser extend in brain metastases arising from breast cancer (Figure 6C). In contrast, CSF-1R expression seemed to be comparable in the various metastases samples and significantly higher than its expression in normal brain tissue (Figure 6D). Expression levels of IL-34 were comparable between metastases samples and healthy tissue although some breast cancer brain metastases display very high expression of this gene (Figure 6E). Finally, recent data revealed a switch between invasion and proliferation in *claudin-low* breast carcinoma cells mediated via an autocrine activation of CSF-1R signalling [28]. Thus, we tested the gene expression of CSF-1 and CSF-1R in primary tumor samples and correlated their expression to the molecular breast cancer subtypes as well as the incidence of metastases. For this purpose 11 microarray datasets of primary tumors of breast cancer patients, with the annotation of time do distant metastasis, were analyzed. All samples were classified regarding their molecular subtype (Figure 6F) and expression of CSF-1 and CSF-1R was analyzed. As illustrated in Figure 6G expression of both genes differs significantly in the 2082 samples, in particular CSF-1R. However, there was no correlation, neither with the molecular subtypes, nor with the incidence of metastasis in these data-sets.

## DISCUSSION

**Figure 7:**
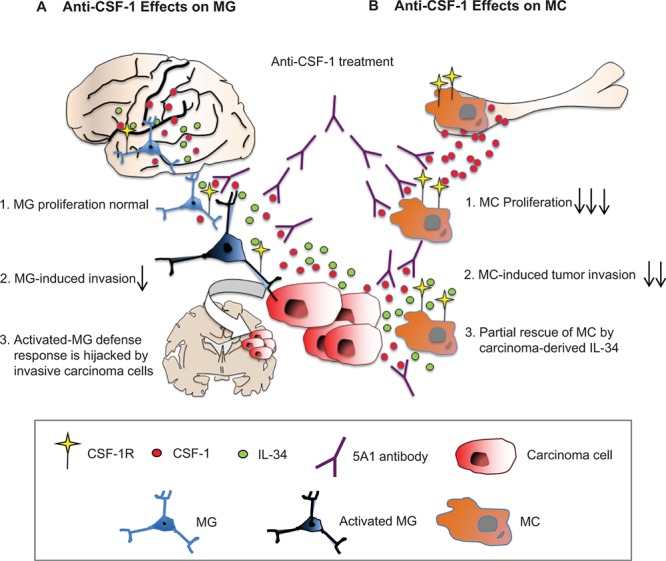
Differential effects of anti CSF-1 treatment on MC and MG **A.** IL-34, CSF-1 and others activate MG to instigate a defense response that is hijacked by invasive tumor cells thus promoting incipient metastasis. **B.** The BM is deprived of IL-34. MC are addicted to CSF-1 and its blockage attenuates MC-induced tumor invasion. However, partial rescue is obtained due to carcinoma-derived IL-34. CSF-1R converges brain-specific-IL-34 and CSF-1 signaling and is therefore potential therapeutic target.

Here we demonstrate that resident MG are not addicted to CSF-1 while MCs are very sensitive to anti-CSF-1 treatment. Furthermore, breast cancer cells are not only a source of CSF-1 as already described, but also a source for IL-34, the alternative ligand for the CSF-1R. This is reflected by its high expression in the metastatic basal-like MDA-MB231 breast cancer cells and in human brain metastases. The great importance of this alternative ligand is underlined not only by the finding that the more aggressive and IL-34-expressing MDA-MB231 cells induced a significant increase in MC proliferation, but also by the fact that IL-34 could partially overcome the effect of anti-CSF-1 on MCs. When analysing IL-34 expression in the bone marrow, as compared with the brain parenchyma, we only detected IL-34 expression in the brain, indicating significant differences in the brain microenvironment (summarized in Figure 7).

Thus, IL-34 expression could participate in an organ- (namely brain-) specific mechanism that contributes to resistance to CSF-1 depletion e.g. in the CSF-1^op/op^ model, where MG are only reduced in numbers [19]. When employing CSF-1 or CSF-1R as a therapeutic target, treatment resistance, particularly of brain metastases, has to be considered. Such resistance may be caused by the production of CSF-1 and IL-34 not only by aggressive carcinoma cells but also by the brain parenchyma itself.

However, although we detected no differences in the viability of MG after anti-CSF-1 treatment, MG-induced invasion of breast cancer cells was reduced following anti-CSF-1 treatment, but to a lower extent than MC-induced invasion. These results could point to a phenotypic shift of MG induced by CSF-1 inhibition as already mentioned [19, 20]. A comparable phenotype shift was recently described in a glioblastoma model where inhibition of the CSF-1R reduced the glioma growth by qualitative but not quantitative effects on MC and MG [29]. Additionally, we analyzed a subset of primary breast cancer patients, and certain patients with brain metastasis of lung and breast cancer and found high CSF-1R, CSF-1 and/or IL-34 expression, respectively. Thus, blocking CSF-1 or IL-34 signaling by inhibition of the common receptor CSF-1R could be an innovative treatment strategy, as shown in the glioma model [29]. Importantly, some CSF-1 or CSF-1R inhibitors are already available and are in clinical trials for the treatment of advanced carcinoma, e.g. LY3022855 (IMC-CS4) (NCT01346358, phase I: metastatic breast and prostate cancer); PLX 3397 (NCT01596751 phase Ib/II: metastatic breast cancer). However, there are also conflicting data demonstrating that treatment with anti-CSF-1R antibodies or inhibitors could lead to increased metastasis via up-regulation of GM-CSF in breast cancer models [30]. In context of these findings and our results, it will be very important to further investigate the effect of anti-CSF-1R treatment on different cell types of the MPS system and to establish predictive biomarkers in cancer patients. Eventually, CSF-1R expression could be a promising candidate as demonstrated here in the primary breast cancer data sets of 2082 patients.

Taken together, our most significant finding is the fundamental difference between MCs and MG, supporting the importance of the new MPS classification and the categorization into macrophages, MCs and DCs [25]. Although in this study only *in vitro* systems were applied, we demonstrate that this could have significant influence on innovative treatment strategies addressing the MPS, in particular in metastasis. Additionally, our findings emphasize the role of resident macrophages and organ-specific defense systems during the crucial step of metastasis, at least in metastatic organs with unique macrophage populations, like liver, lung and brain. Importantly, once assuming the *parallel progression model* of metastasis, organ-specific defense systems represent one of the most important targets to prevent metastatic outgrowth of disseminated carcinoma cells. Thus, strengthening the organ-specific defense systems may be a very potent therapeutic strategy to deplete incipient metastatic cells at distant organs during the treatment of primary carcinomas.

## MATERIALS AND METHODS

### Cells, media and reagents

If not indicated otherwise, all substances were purchased from Sigma (Munich, Germany). The human breast cancer cell lines MCF-7 and MDA-MB231 were obtained from the American Type Culture Collection (Rockville, USA). The human cell lines were cultivated in RPMI-1640 medium (PAA, Cölbe, Germany) supplemented with 10% heat inactivated fetal bovine serum (FCS; Sigma, Munich, Germany). The BALB/C murine breast cancer cell line 410.4, obtained from the American Type Culture Collection (Rockville, USA), was cultured in DMEM + 10% FCS. The anti-CSF-1 antibody was obtained from Novartis (Basel, Switzerland). Recombinant IL-34 was obtained from BioLegend (Fell, Germany) and a concentration of 100 ng/ml was used in the experiments.

### Isolation of MCs

Murine MCs were isolated as described previously [31]. Briefly, femurs of 8-12 week old NMRI mice were flushed with growth medium (DMEM (Biochrome, Berlin, Germany) + 10% heat inactivated FCS (Sigma, Munich, Germany), 5% heat inactivated NHS (Gibco, Darmstadt, Germany), 30% L929 conditioned medium, 2 mM L-glutamine, 0,01 mM sodium pyruvate, 0,05 mM 2-mercaptoethanol, 100 U/ml penicillin and 100 mg/ml streptomycin) and cultivated overnight in cell culture dishes (Nunc, Wiesbaden, Germany). Non-adherent cells were collected and cultured for 6 days in non-coated culture dishes (Sarstedt, Nümbrecht, Germany) in growth medium. For experiments MCs were cultured in DMEM + 10% heat inactivated FCS + 15% L929 conditioned medium. L929 conditioned medium contains CSF-1 and was prepared as previously described [32].

### Isolation of murine MG

Primary MG cell cultures from newborn (P0) NMRI mice were prepared and cultured as previously described [33]. After 10-14 days, MG cells were plated in cell culture plates or inserts and used 24 h later for experiments.

### Metabolism assay

Cell metabolism was analysed by measurement of MTT (2,3-diphenyl-5-methyltetrazolium chloride; Sigma, Munich, Germany) conversion according to standard procedures. Briefly, cells were incubated with 0,5 mg/ml MTT for 4 h at 37°C. Subsequently cells were lysed (5% formic acid in isopropanol + DMSO (2:1)) and optical density was measured at 550 nm. Cells were treated with anti-CSF-1 (5A1) for 96 h before measuring MTT reduction. For co-culture assays 5 × 10^4^ MCs per well were seeded in 500 μl medium and 1 × 10^5^ tumor cells were seeded in a transwell insert (Nunc, Wiesbaden, Germany) containing 500 μl medium. After at least 1 h of incubation, to allow the cells to attach, the transwell insert was transferred into the well containing MCs. Co-cultures were performed for 96 h, before transwells were discarded and MTT measurement was performed for MCs.

### Proliferation assay

Cell proliferation assays were performed using the xCELLigence RTCA DP system (Roche, Mannheim, Germany). A cell density of 1 × 10^3^ (MDA-MB231), 4 × 10^4^ (MCF-7, MCs) or 8 × 10^4^ MG cells per well were plated and proliferation/morphology was analyzed for 48 h in quadruplets. Treatment with 5A1 was performed once at time point 0 h.

### Calcein-AM / propidium iodid (PI)-staining

Cell viability staining was performed by calcein-AM / PI double staining. A cell density of 1 × 10^5^ cells (MCs, MG) were seeded on round cover slips (12 mm diameter) and were treated with 2,5 μg/ml 5A1 for 24 h, 48 h, 72 h and 96 h, respectively. For calcein-AM / PI double staining, cells were incubated in 1 μM calcein-AM (Sigma-Aldrich, Seelze, Germany) plus 1 μM PI (Sigma-Aldrich, Seelze, Germany) for 30 min at 37°C in the dark. Subsequently, cells were fixed with 4% paraformaldehyde for 15 min before placing cover slips upside down on an object slide using mounting medium (Dako, Hamburg, Germany). Fluorescence signals were analysed with Leica DM500B (Leica, Wetzlar, Germany).

### Extracellular matrix (ECM)-based migration assay

Migration assays were performed as previously described [13]. Migration was analyzed by measuring the area covered by tumor cells after 48 h using the Axiovert 200M microscope and the Axiovision Rel.4.6.3 Software (Zeiss, Göttingen, Germany). 25 μg/ml 5A1 was added at time point 0 h.

### Microinvasion assay

Invasion was measured in a modified Boyden chamber as described previously [27]. Briefly, 1 × 10^5^ MCF-7 or 410.4 breast cancer cells were seeded into the upper well of the chamber in 500 μl medium, the lower well was filled with 1 ml of the same medium. For co-culture experiments 1,5 × 10^5^ MCs or 2 × 10^5^ MG were seeded in transwell inserts (Nunc, Wiesbaden, Germany) in 500 μl medium. The medium used was either DMEM + 10% FCS (co-cultures with MG) or DMEM + 10% FCS + 15% L929 conditioned medium (co-cultures with MCs). The transwells were inserted into the upper well of the Boyden chamber and anti-CSF-1 was added. After 96 h the floating and adherent carcinoma cells in the lower well were removed and counted. All experiments were performed at least in triplicate.

### RNA isolation

RNA from tissue was isolated with a modified Trizol (Invitrogen, Darmstadt, Germany) method incorporating a DNaseI (Roche, Mannheim, Germany) digestion step. RNA from cells was isolated using the “High Pure RNA isolation kit” (Roche, Mannheim, Germany). Reverse transcription was performed with the iScript Master Mix (BioRad, Munich, Germany).

### qRT-PCR

Quantitative RT-PCR was performed as previously described [34]. The following mRNA specific, intron-spanning primers were used: mmCsf-1, mmCsf-1r, mmIl-34, hsCSF-1, hsCSF-1R and hsIL-34 (sequences see Supplementary Table S1). All qRT-PCRs were performed using the HT 7900 system (Applied Biosystems, Darmstadt, Germany). Gene expression was analysed by using the SDS Software Version 2.4 (Applied Biosystems) normalizing the expression to two housekeeping genes, mmTbp/mmGapdh and hsHPRT1/hsGNB2L1.

### Western blot

Cells were lysed and homogenized in RIPA lysis buffer (150 mM NaCl/0,1% SDS/0,5% Na-deoxycholate/1% Triton X-100/50 mM Tris, pH 7,2). Up to 30-60 μg of total protein were subjected to SDS-PAGE (10%) and blotted onto a nitrocellulose membrane (Amersham Biosciences). Ponceau S staining was used as loading control. Membranes were incubated with antibodies specific to AKT (#9272, Cell Signaling), pAKT (#9275, Cell Signaling), S6 (#2217, Cell Signaling), pS6 (#2211, Cell Signaling) and anti-human-CSF-1 (5H4, Basel, Novartis). Signals were detected with ECL Prime (Amersham Biosciences).

### Statistical analysis for the experimental procedures

Using the Student's *t*-test the significance of the differences between groups in the qRT-PCR, Boyden chamber, MTT experiments etc. was tested and *p*-values < 0.05 were considered significant.

### Microarray datasets and bioinformatics

Three public microarray datasets were retrieved from NCBI Gene Expression Omnibus (GEO) [35] data repository comprising 15 brain metastases from primary breast cancers (GSE14017), 19 brain metastasis samples of primary adenocarcinomas of the lung (GSE14108) and 3 control brain samples (GSE7905). As these datasets are profiled on different platforms, first each dataset was summarized on gene level and then combined into one matrix which was quantile normalized. Another retrieved batch of 11 GEO datasets (GSE20685, GSE19615, GSE17907, GSE16446, GSE17705, GSE2603, GSE11121, GSE7390, GSE6532, GSE6532) comprised together 2082 breast cancer patients with annotation of time to distant metastasis, which occurred in 512 patients. The data were profiled on Affymetrix Human Genome U133A and U133 Plus 2.0 arrays. First, all datasets were preprocessed using RMA algorithm [36], then the data were combined together on the bases of HG-U133A array probes and quantile normalized. Breast cancer molecular subtypes of 2082 patients were identified by fitting a single sample predictor as implemented in *genefu* r-package [37, 38] with *pam50* intrinsic genes list option for subtype prediction [39]. All analyses were performed using the free statistical software R (version 2.15.1; http://www.r-project.org).

## SUPPLEMENTARY FIGURES, TABLES LEGENDS


