# Supplementary material for: Anti-CSF-1 treatment is effective to prevent carcinoma invasion induced by monocyte-derived cells but scarcely by microglia
Source: Oncotarget. 2015 May 12;6(17):15482–93. doi: 10.18632/oncotarget.3855 (PMC4558165; doi:10.18632/oncotarget.3855)
Supplement: Supplementary file 1 [file oncotarget-06-15482-s001.pdf]

SUPPLEMENTARY FIGURES, TABLES LEGENDS

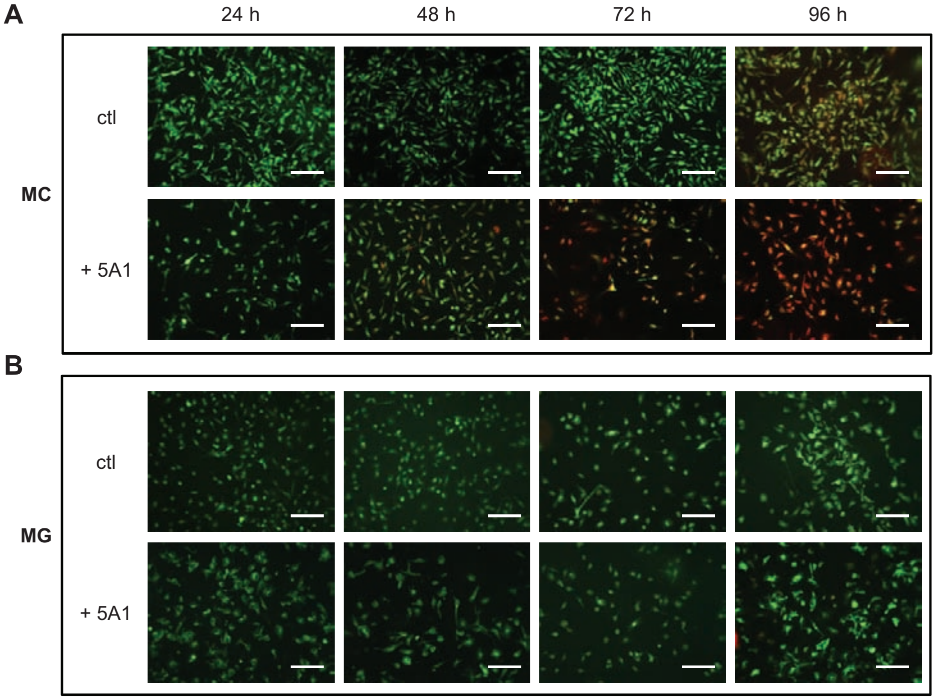

Supplementary Figure S1: 5A1 induces apoptosis of MC but not MG. MCs **A.** and MG **B.** were treated with 2,5 µg/ml 5A1 and calcein-AM / PI-staining was performed after 24 h, 48 h, 72 h and 96 h of incubation, respectively, for untreated (ctl) and treated (+5A1) cells. Scale bars indicate 0.1 mm. Experiments were performed in triplicates, shown is one representative result.

Supplementary Table S1: Primer Sequences

Gene	fwd	rev
hsCSF-1	GGAGACCTCGTGCCAAATTA	CGCATGGTGTCCTCCATTAT
hsCSF-1R	CGACTATAAGAACATCCACCT	CATTTGAAGAAGTGGAGACAG
hsGNB2L1	AACCCTATCATCGTCTCCT	CAATGTGGTTGGTCTTCAG
hsHPRT1	TATGCTGAGGATTTGGAAAGG	CATCTCCTTCATCACATCTCG
hsIL-34	CCCATCCTGGAAGTACCT	CATTCAAGAGGGACAACAC
mmCsf-1	GCGCTTTAAAGACAACACCC	ATGGAAAGTTCGGACACAGG
mmCsf-1r	CACCATCCACTTGTATGTC	CTCAACCACTGTCACCTC
mmGapdh	CATCTTGGGCTACACTGAG	CTGTAGCCGTATTCATTGTC
mmIl-34	TGGCTTTGGGAAACGAGAAT	CCCTCATAAGGCACAGCAAT
mmTbp	TTCCCAGCTAAGTTCTTAGAC	CCAGGAAATAATTCTGGCTC
